# The impact of blood pressure targets on haemodynamics in patients with cardiogenic shock caused by infarction-related out-of-hospital cardiac arrest: a substudy of the BOX-trial

**DOI:** 10.1093/ehjacc/zuag045

**Published:** 2026-05-11

**Authors:** Sanne ten Berg, Rasmus P Beske, Johannes Grand, Henrik Schmidt, Jesper Kjærgaard, Jacob E Møller, Mathilde M B Jessen, José P S Henriques, Christian Hassager

**Affiliations:** Department of Cardiology, Amsterdam University Medical Center, University of Amsterdam, Amsterdam Cardiovascular Sciences, Amsterdam, The Netherlands, Meibergdreef 9, 1105 AZ, Amsterdam; Department of Cardiology, The Heart Center, Copenhagen University Hospital, Rigshospitalet, 9 Blegdamsvej, DK2100 Copenhagen East, Copenhagen, Denmark; Department of Cardiology, The Heart Center, Copenhagen University Hospital, Rigshospitalet, 9 Blegdamsvej, DK2100 Copenhagen East, Copenhagen, Denmark; Department of Anesthesiology and Intensive Care, Odense University Hospital, J. B. Winsløws Vej 45000 Odense C, Odense, Denmark; Department of Cardiology, The Heart Center, Copenhagen University Hospital, Rigshospitalet, 9 Blegdamsvej, DK2100 Copenhagen East, Copenhagen, Denmark; Department of Cardiology, The Heart Center, Copenhagen University Hospital, Rigshospitalet, 9 Blegdamsvej, DK2100 Copenhagen East, Copenhagen, Denmark; Department of Anesthesiology and Intensive Care, Odense University Hospital, J. B. Winsløws Vej 45000 Odense C, Odense, Denmark; Department of Anesthesiology and Intensive Care, Odense University Hospital, J. B. Winsløws Vej 45000 Odense C, Odense, Denmark; Department of Cardiology, Amsterdam University Medical Center, University of Amsterdam, Amsterdam Cardiovascular Sciences, Amsterdam, The Netherlands, Meibergdreef 9, 1105 AZ, Amsterdam; Department of Cardiology, The Heart Center, Copenhagen University Hospital, Rigshospitalet, 9 Blegdamsvej, DK2100 Copenhagen East, Copenhagen, Denmark

**Keywords:** Cardiogenic shock, Cardiac arrest, Myocardial infarction, Haemodynamics

## Abstract

**Aims:**

Noradrenaline is frequently used to increase mean arterial pressure (MAP) in patients with cardiogenic shock caused by infarction-related out-of-hospital cardiac arrest (OHCA-AMICS). This Blood pressure and Oxygenation-Targets after OHCA (BOX)-trial subanalysis investigates the impact of different noradrenaline-mediated MAP-targets on overall haemodynamics and outcomes in OHCA-AMICS patients.

**Methods and results:**

The BOX-trial compared randomized, double-blinded MAP-targets of 63 mmHg (low-target) and 77 mmHg (high-target) in comatose OHCA patients. For this subanalysis, patients meeting eligibility criteria for AMICS according to the ongoing NORshock trial were included (percutaneous coronary intervention, hypotension, signs of hypoperfusion, and <30 min to return of spontaneous circulation). Haemodynamic parameters assessed were cardiac index (CI) and pulmonary capillary wedge pressure (PCWP). Markers of tissue perfusion comprised mixed venous oxygen saturation (SvO_2_) and lactate. Clinical endpoints included 365-day mortality, renal replacement therapy (RRT), and neurological outcomes. Within the cohort of 258 patients (low-target *n* = 132, high-target *n* = 126), mean age was 63 (±11) years, 84% were male, and patient characteristics were well balanced between groups. The high-target group received significantly higher doses of noradrenaline. CI increased over time in both groups (low-target: 1.50→2.88; high-target: 1.77→2.92 L/min/m^2^). Despite 8–19% higher CIs during the first 24 h, the high-target group consistently exhibited higher PCWPs. SvO_2_ was higher in the high-target group. Lactate levels were comparable and, on average, normalized in both groups within 6 h. Mortality, need for RRT, and neurological outcomes did not differ significantly.

**Conclusion:**

In OHCA-AMICS patients, higher noradrenaline-mediated MAP increased CI and SvO_2_ but did not enhance tissue perfusion or clinical outcomes, and were associated with higher left-sided filling pressures.

## Graphical Abstract

**Graphical Abstract:**
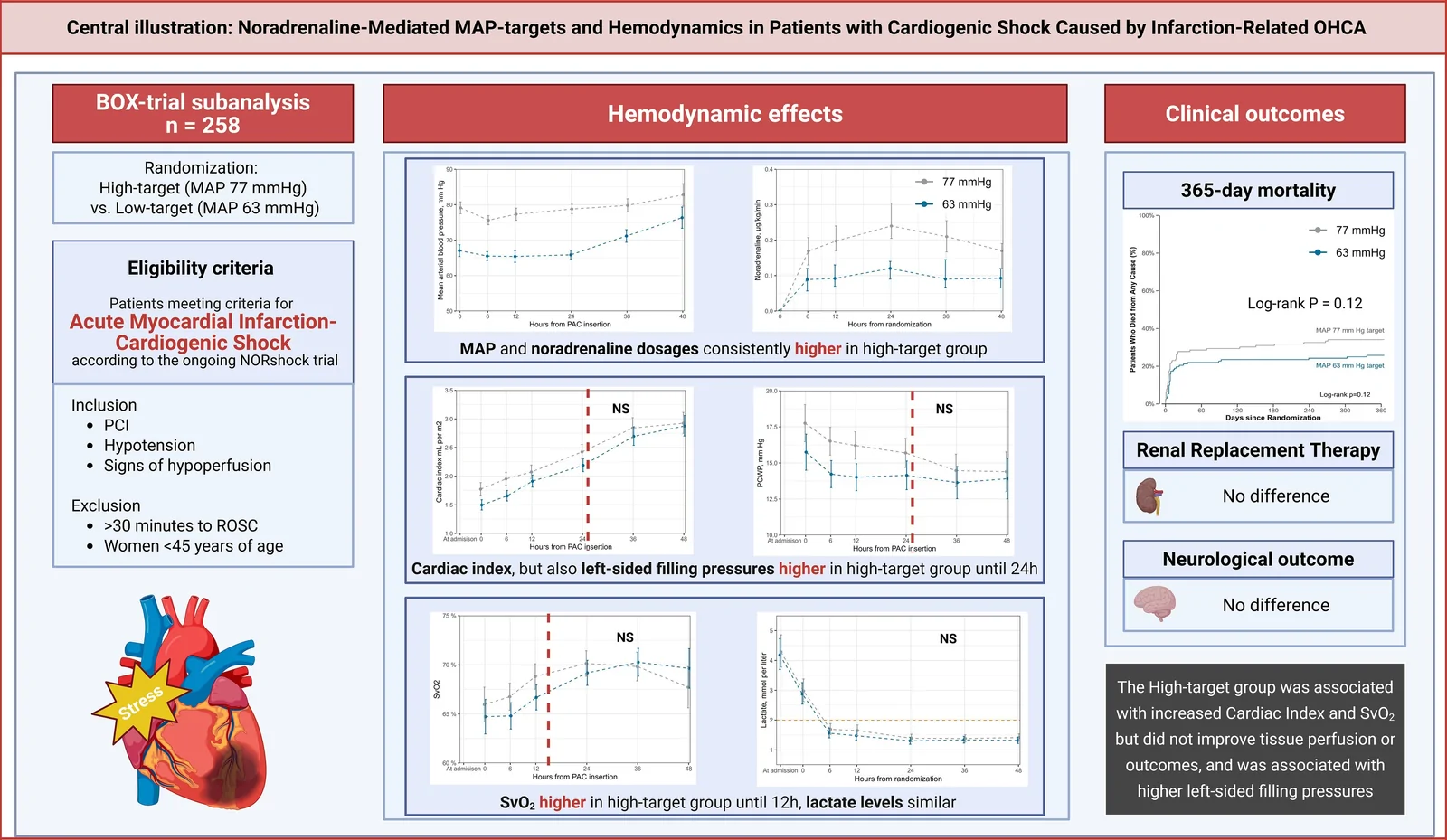

## Introduction

Hypotension is common in the post-resuscitation phase following out-of-hospital cardiac arrest (OHCA), which can be caused by either myocardial infarction, post-cardiac arrest myocardial dysfunction, systemic vasodilation, sedative medication, or a combination thereof.^1^

Phenotypes of OHCA and cardiogenic shock (CS) patients often overlap, as both present with hypotension, high lactate levels, and low cardiac indices (CIs).^2–4^ Noradrenaline is often used to increase blood pressure through alpha-receptors, and it also, to a lesser extent, exerts a positive inotropic and chronotropic effect via beta-receptors.^5,6^ However, noradrenaline increases afterload, may increase myocardial oxygen consumption and decrease myocardial blood flow, and stimulates lactate production, even in the absence of hypoxia.^5,7–11^ Furthermore, vasoconstriction may restore macrovascular haemodynamics but might mask microvascular flow derangements.^12^

These haemodynamic effects of noradrenaline may contribute to the findings observed in the Blood pressure and Oxygenation Targets after OHCA trial (BOX-trial) that compared targeted MAPs of 63 mmHg (low-target group) vs. 77 mmHg (high-target group).^13^ Not surprisingly, higher noradrenaline dosages were administered to the high-target group. A subanalysis thereof demonstrated that lactate levels were higher in the high-target group, despite significantly higher CIs.^14^

The effect of MAP and associated noradrenaline dosages on haemodynamics and clinical outcomes has not been fully established in CS patients. To address this knowledge gap, we analysed BOX-trial patients who would have met the eligibility criteria for the ongoing NORshock trial, allowing for understanding of haemodynamic responses in a population of resuscitated acute myocardial infarction (AMI)-related CS-qualifying patients. The hypothesis of the ongoing NORshock trial is that a lower blood pressure target (MAP 50–55 mmHg) will lead to better outcomes compared with standard of care in patients with AMICS.^15^ In this trial, AMICS patients undergoing percutaneous coronary intervention (PCI) are included.

Our primary objective was to assess the impact of blood pressure targets and associated noradrenaline dosages on overall haemodynamics in patients fulfilling clinical criteria for CS, caused by infarction-related OHCA. For the purposes of this subanalysis, these patients will hereafter be referred to as OHCA-AMICS patients.

## Methods

### Study design and patient population

The BOX-trial was a multicentre, investigator-initiated randomized controlled trial (RCT) that compared targeted MAPs of 63 and 77 mmHg using a double-blind approach.^13,16^ A total of 789 adult patients who remained comatose after OHCA of presumed cardiac origin were included at two tertiary cardiac centres in Denmark between March 2017 and December 2021. The trial was registered at clinicaltrials.gov with the identifier NCT03141099, and the primary outcome was published in 2022.^13^

### Intervention

At the intensive care unit (ICU), all participants underwent invasive blood pressure monitoring using a patient-specific module (M1006B Invasive Blood Pressure Module, Philips). For the purposes of the trial, these devices were calibrated so that the displayed blood pressure was either 10% lower or higher than the true value, depending on the group assignment. This modification ensured that all patients were targeted towards a MAP of 70 mmHg, though they were actually targeted towards a MAP of 63 and 77 mmHg, respectively. The allocation of blood pressure targets was concealed from clinical staff, investigators, patients, and those assessing outcomes.

### Study procedures

The blood pressure intervention began immediately upon patient inclusion and was maintained for the duration of invasive arterial blood pressure monitoring. As an initial step, a central venous pressure (CVP) of 10 mmHg was achieved with volume resuscitation. If not sufficient, noradrenaline was administered as the initial vasopressor at 0.01 mcg/kg/min with incremental increase of 0.01 mcg/kg/min if deemed necessary to achieve a MAP of 70 mmHg. Should the MAP target not be achieved with noradrenaline doses exceeding 0.2 mcg/kg/min, the treating physician could either further increase the noradrenaline dose or add dopamine. If this was insufficient to maintain adequate cardiac output, alternative inotropic agents could be considered.

### Study population

Details on the inclusion and exclusion criteria of the BOX-trial have been described previously.^13^ In this subanalysis of the BOX-trial, patients who were eligible for inclusion in the ongoing NORshock trial were included (see Supplementary material online, *Table S1)*.^15^ In line with these criteria, patients were eligible if they had presented with an AMI, not restricted to ST-segment-elevation MI, and had undergone PCI. Additionally, patients were required to have clinical signs of CS, defined as hypotension with a systolic blood pressure below 90 mmHg for over 30 min, or the use of vasopressors to maintain that pressure and signs of impaired organ perfusion such as an elevated lactate (>2.0 mmol/L) or altered mental status. Patients were excluded if they had been resuscitated for more than 30 min, had a mechanical cause of CS or were women under 45 years of age. Patients who had been in shock for over 12 h or required extracorporeal cardiopulmonary resuscitation are excluded in the NORshock trial, which is in line with the exclusion criteria of the BOX-trial.

Classifications based on the Society for Cardiovascular Angiography and Intervention (SCAI) criteria were obtained from a previously published subanalysis, in which patients were classified according to a slightly modified version of the SCAI classification, where OHCA did not automatically qualify as stage E.^17^

### Haemodynamic measurements, tissue perfusion, and metabolic status

Regarding the primary haemodynamic measurements, noradrenaline dosages, vasopressor-inotropic scores, CIs, CVP, systemic vascular resistance (SVR), and pulmonary capillary wedge pressures (PCWPs) were compared between groups. Markers of tissue perfusion and metabolic status included lactate levels, pH, base excess (BE), and mixed venous oxygen saturation (SvO_2_).

As soon as feasible after randomization, a standard commercially available balloon-tipped pulmonary artery catheter (PAC) was inserted in 93% of the whole and current cohort.^14^ Comprehensive haemodynamic assessments were performed, including cardiac output measurements by thermodilution, SvO_2_, and PCWP measurements. These measurements were conducted at the time of PAC insertion and subsequently at 6, 12, 24, 36, and 48 h. For obvious reasons, analyses and plots of PAC measurements over time were initiated from the time of PAC insertion.

Infusion dosages of noradrenaline and other vasoactive agents were recorded electronically. The Vasoactive-Inotropic Score (VIS) was calculated according to the following formula: dopamine (µg/kg/min) + dobutamine (µg/kg/min) + 100 × adrenaline (µg/kg/min) + 100 × noradrenaline (µg/kg/min) + 10 × milrinone (µg/kg/min) + 50 × levosimendan (µg/kg/min) + 10 000 × vasopressin (units/kg/min).

Laboratory markers of tissue perfusion and metabolic status had been collected at predefined time points and were supplemented with all additional values available from the electronic patient records.

### Clinical endpoints

Clinical endpoints comprised all-cause mortality at 365 days, need for renal replacement therapy (RRT), and neurological scores. In the BOX trial, neurological outcome was part of the primary composite endpoint together with mortality, whereas in the NORshock trial, neurological outcome will be analysed as a secondary endpoint. For this analysis, both the cerebral performance category scale (CPC) and modified ranking scale (MRS) scores were used, with higher categories indicating more severe disability. As the primary endpoint of the NORshock trial is a composite endpoint of 30-day mortality and RRT, an exploratively composite endpoint of all-cause 365-day mortality and RRT during index hospitalization was considered.

### Ethics

The BOX-trial received approval from the Regional Ethics Committee of the Capital Region of Denmark (Approval ID: H-16033436) and the Danish Medicines Agency (Approval ID: RH-2016-373).

### Statistical analyses

Continuous variables were reported as mean values with standard deviations (SDs) when the data followed a normal distribution, or as medians with interquartile ranges (IQRs) when non-normally distributed. Differences between groups for continuous variables were evaluated using either the *t*-test or the Mann–Whitney *U* test, depending on the distribution of the data. For the primary haemodynamic endpoints, which were measured repeatedly over time, linear mixed-effects models were used to account for within-subject correlations. Group differences were evaluated at the pre-specified time points. However, for noradrenaline and VIS, residual diagnostics from the mixed model, including Q–Q plots, indicated substantial deviations from normality that could not be corrected through transformation. Therefore, noradrenaline dose and VIS were summarized as medians with bootstrapped 95% confidence intervals (CIs), and group differences were assessed using the Mann–Whitney *U* test. Categorical variables were described by frequencies and percentages, and comparisons between groups were performed using the chi-squared or the Fisher’s exact tests.

The clinical outcome of 365-day mortality was plotted with the Kaplan–Meier method and analysed with the log-rank test. Results were presented as hazard ratios (HRs) and corresponding 95% CIs. Other clinical endpoints, such as the need for RRT and neurological outcomes (CPC and mRS scores), were analysed using binary and ordinal logistic regression, respectively, and are presented as odds ratios (ORs). The combined endpoint of 1-year mortality or need for RRT during index hospitalization was compared between groups.

Statistical significance of all analyses was defined as a *P*-value <0.05. All analyses were performed with R statistical software (version 4.5.0, R Core Team, 2025).

## Results

### Patient selection and study cohort

In total, 789 patients were included in the BOX-trial. For the present substudy, 258 individuals were included based on the in-and exclusion criteria of the NORshock trial (see Supplementary material online, *Table S2*).

*Study cohort of NORshock-eligible patients* Within the cohort of 258 patients, 132 patients were classified into the low-target group and 126 to the high-target group (*Table 1*). All baseline characteristics, comorbidities and admission parameters were comparable between the two groups. The mean age was 63 years (±11) and the majority was male (*n* = 217, 84%). Notably, the proportions of witnessed cardiac arrest (*n* = 218, 84%) and bystander CPR (*n* = 208, 82%), as well as the median time to return of spontaneous circulation [16 min (11–22)], did not differ significantly. Also, left ventricular ejection fraction (LVEF) [35% (25–45)], median lactate levels [4.4 mmol/L (2.4–7.6)] and glucose levels [13.3 mmol/L (10.5–16.8)] upon arrival were similar between groups. There were no statistically significant differences in SCAI classifications.

**Table 1:** Study cohort baseline characteristics

Characteristic	Overall	Low	High	*P*-value
	*n* = 258	*n* = 132	*n* = 126	
**Baseline characteristics**				
Age, years, mean (SD)	63 (11)	62 (12)	63 (11)	0.54^a^
Sex, male—*n* (%)	217 (84%)	110 (83%)	107 (85%)	0.73^b^
Body mass index, *n* (%)				0.24^c^
Underweight, BMI < 18.5	1 (0.4%)	0 (0%)	1 (0.8%)	
Normal weight, BMI 18.5–24.9	99 (40%)	55 (43%)	44 (36%)	
Overweight, BMI 25–29.9	94 (38%)	50 (39%)	44 (36%)	
Obese, BMI ≥ 30	55 (22%)	23 (18%)	32 (26%)	
**Co-morbidities**				
Heart failure, no. (%)	18 (7.0%)	7 (5.3%)	11 (8.8%)	0.27^b^
Diabetes, no. (%)	35 (14%)	17 (13%)	18 (14%)	0.74^b^
Ischemic heart disease, no. (%)	46 (18%)	18 (14%)	28 (22%)	0.07^b^
**OHCA characteristics**				
Witnessed arrest, no. (%)	218 (84%)	110 (83%)	108 (86%)	0.60^b^
Bystander cardiopulmonary resuscitation, no. (%)	208 (82%)	110 (84%)	98 (80%)	0.45^b^
Time to ROSC, median (IQR)	16 (11, 22)	15 (10, 20)	17 (12, 24)	0.10^d^
**Admission characteristics**				
LVEF at arrival, median (IQR)	35 (25, 45)	35 (25, 45)	35 (25, 45)	0.41^d^
Lactate at arrival, mmol/L, median (IQR)	4.4 (2.4, 7.6)	4.4 (2.7, 6.9)	4.4 (2.3, 7.9)	0.88^d^
Glucose at arrival, mmol/L, median (IQR)	13.3 (10.5, 16.8)	13.4 (10.8, 16.8)	13.2 (10.4, 16.8)	0.50^d^
SCAI class at admission, *n* (%)				0.40^b^
SCAI class B/C	82 (32%)	47 (36%)	35 (28%)	
SCAI class D	88 (34%)	42 (32%)	46 (37%)	
SCAI class E	88 (34%)	43 (33%)	45 (36%)	
ST-segment elevation ECG, no. (%)	182 (71%)	93 (72%)	89 (71%)	0.95^b^

BMI, body mass index; ECG, electrocardiogram; IQR, interquartile range; LVEF, left ventricular ejection fraction; OHCA, out-of-hospital cardiac arrest; ROSC, return of spontaneous circulation; SCAI, Society for Cardiovascular Angiography and Intervention; SD, standard deviation.

^a^Welch two-sample *t*-test.

^b^Pearson’s chi-squared test.

^c^Fisher’s exact test.

^d^Wilcoxon rank sum test.

### Haemodynamics

**Figure 1:**
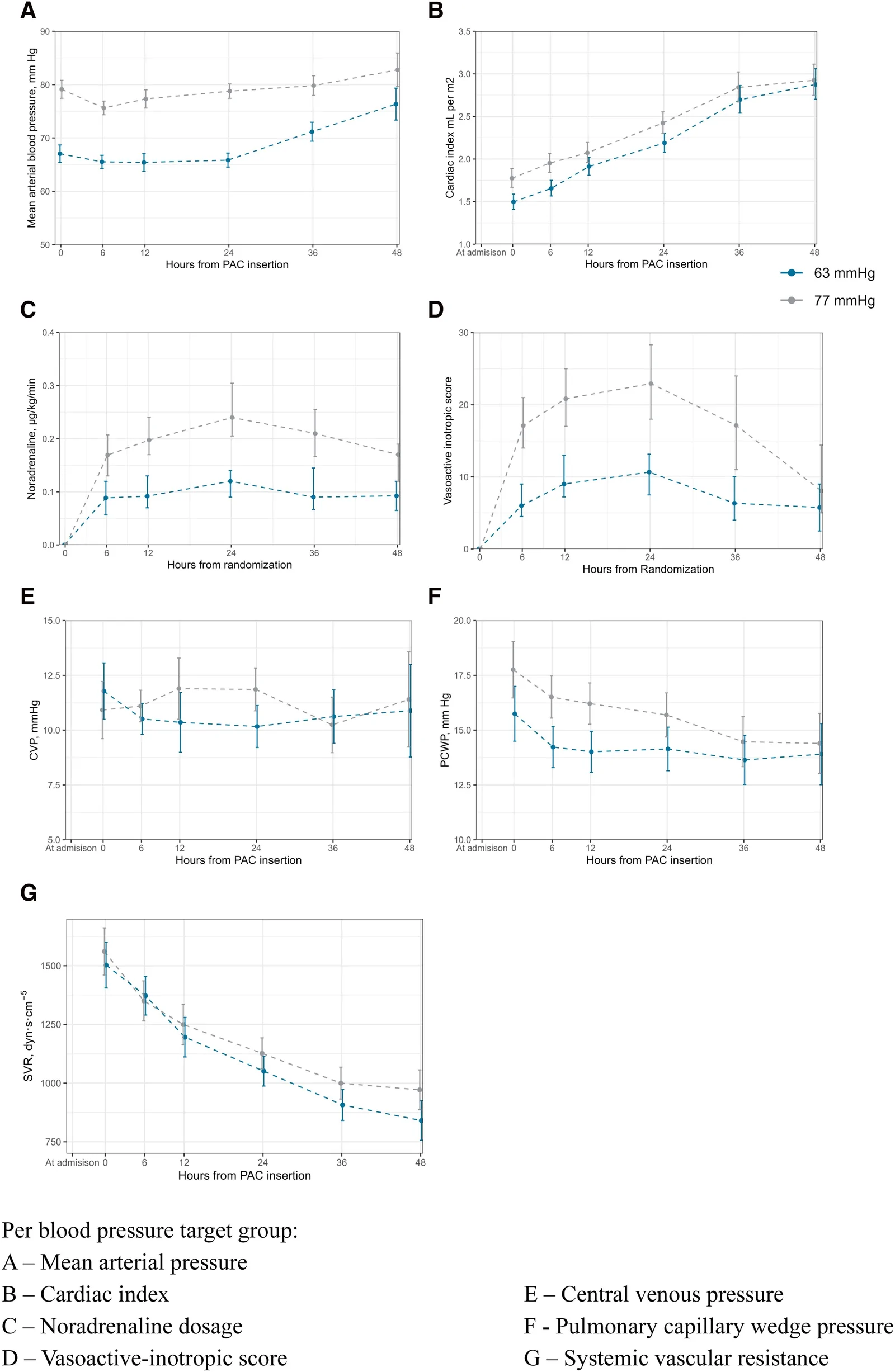
Haemodynamics. Per blood pressure target group: (*A*)—Mean arterial pressure (*B*)– Cardiac index (*C*)—Noradrenaline dosage (*D*)—Vasoactive-inotropic score (*E*)—Central venous pressure, (*F*)—Pulmonary capillary wedge pressure, (*G*)—Systemic vascular resistance.

The achieved MAPs differed significantly between the low- and high-target groups at all assessed time points (*Figure 1,*  Supplementary material online, *Table S3*). The high-target group received significantly more fluids during the first 2 days of admission (mean difference 728 mL, 95%-CI 82–1373 mL; *P* = 0.027). Also, noradrenaline dosages and VIS were consistently higher in the high-target group throughout the observation period. Median time from collapse to placement of PAC was 4 h (IQR 3–5 h). CI increased over the first 48 h in both groups (low-target: 1.50→2.88; high-target: 1.77→2.92 L/min/m^2^). The high-target group had higher CIs early after PAC insertion; 19% higher at 0 h, 18% higher at 6 h, and 8–11% higher at 12–24 h, while differences were small and non-significant after 24 h. In general CVP was similar between the groups. Until 24 h, the SVR was comparable, but after 24 h SVR became significantly higher in the high-target group. PCWP values were higher in the high-target group during the first 24 h, subsequently dropping below 15 mmHg.

### Markers of tissue perfusion and metabolic status

**Figure 2:**
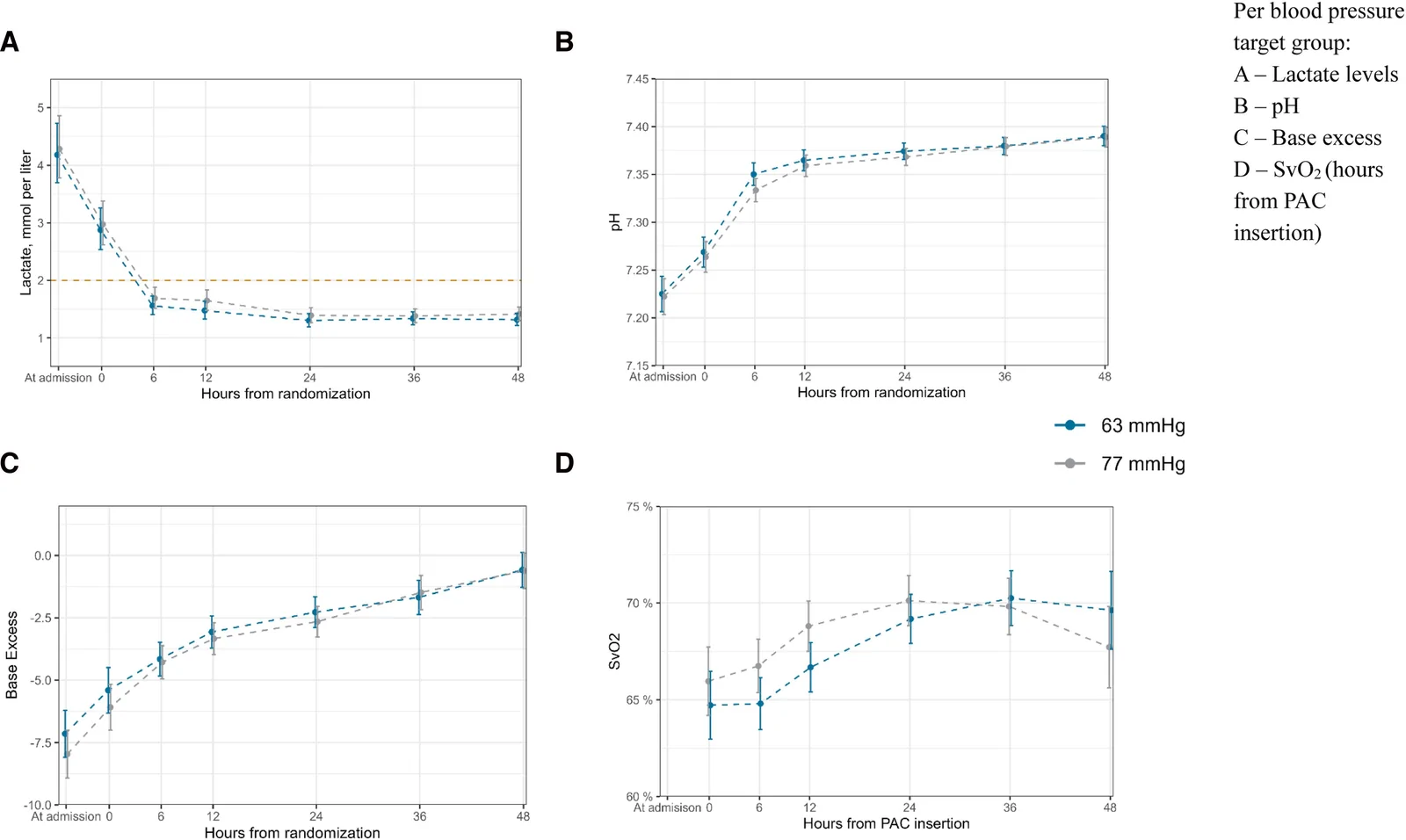
Tissue perfusion and metabolic status. Per blood pressure target group: (*A*)—Lactate levels (*B*)—pH. (*C*)—Base excess. (*D*)—SvO_2_ (hours from PAC insertion).

No significant differences in lactate levels, pH, or BE between groups were observed at any time point (*Figure 2,*  Supplementary material online, *Table S2*). At admission, median lactate levels in both groups were above 4 mmol/L, but rapidly declined to below 3 mmol/L, and normalized within the first 6 h. The estimated mean SvO_2_ was within 60–80% at all time points. However, SvO_2_ was significantly higher in the high MAP group at 6 and 12 h following PAC insertion, but was comparable between groups at all other time points.

### Clinical outcomes

**Figure 3:**
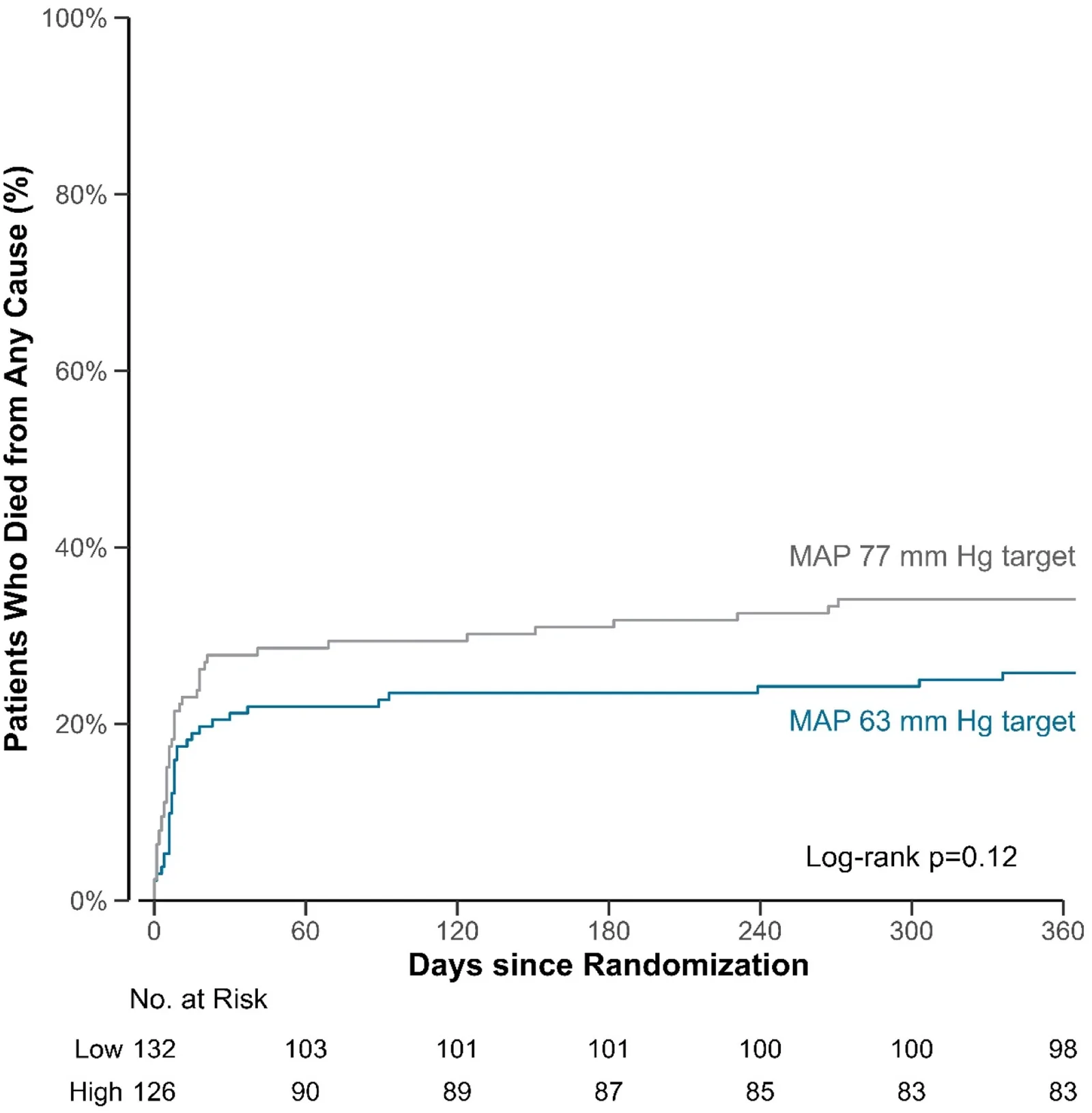
Kaplan meier curves for all-cause mortality at one year.

At 1 year, 34 patients (26%) had died in the low-target group and 43 patients (34%) had died in the high-target group (*Table 2*). The HR for mortality was 0.70 (95% CI, 0.45–1.10). *Figure 3* presents the corresponding Kaplan-Meier curves (pLogrank = 0.12).

**Table 2:** Clinical outcomes

Characteristic	Overall	Low	High	Effect estimates with 95% CI, *P*-value
	*n* = 258	*n* = 132	*n* = 126	
One-year all-cause mortality, *n* (%)	77 (30%)	34 (26%)	43 (34%)	HR 0.70 (0.45–1.10)
Renal replacement therapy, *n* (%)	16 (6.2%)	8 (6.1%)	8 (6.3%)	OR 0.95 (0.34–2.67)
Highest cerebral performance category scale at 1 year, *n* (%)				OR 0.76 (0.46–1.24)
1	153 (61%)	81 (63%)	72 (58%)	
2	16 (6.3%)	10 (7.8%)	6 (4.8%)	
3	5 (2.0%)	3 (2.3%)	2 (1.6%)	
4	1 (0.4%)	0 (0%)	1 (0.8%)	
5	77 (31%)	34 (27%)	43 (35%)	
Highest modified ranking scale at 1 year, *n* (%)				OR 0.74 (0.47–1.16)
0	83 (33%)	49 (39%)	34 (27%)	
1	58 (23%)	22 (17%)	36 (29%)	
2	25 (10.0%)	15 (12%)	10 (8.1%)	
3	5 (2.0%)	5 (3.9%)	0 (0%)	
4	3 (1.2%)	2 (1.6%)	1 (0.8%)	
6	77 (31%)	34 (27%)	43 (35%)	

CI, confidence interval; HR, hazard ratio; OR, odds ratio.

The use of RRT did not differ significantly between the low and high blood pressure target groups, with eight patients (6.1%) requiring RRT in the low group and eight patients (6.3%) in the high group [OR 0.95 (0.34–2.67)]. The combined endpoint of mortality at 1 year or RRT during index hospitalization occurred in 37 (28%) and 45 (36%) in the low and high blood pressure target groups, respectively (relative risk 0.78 [95% CI, 0.55–1.12]). Neurological outcomes, as assessed by the highest CPC and mRS score at 1 year, were statistically comparable between groups, but numerically in favour of the low-target group (CPC OR 0.76 [0.46–1.24], mRS OR 0.74 [0.47–1.16]).

## Discussion

In this substudy from the BOX-trial, including OHCA-AMICS patients fulfilling clinical criteria for CS at the time of inclusion, the high-target group exhibited significantly higher MAPs, CIs, and SvO_2_ at specific time points, whereas tissue perfusion as reflected by lactate levels was similar between groups at all time points. Also, PCWP was higher in the high-target group, indicating greater cardiac and pulmonal stress. Clinical outcomes, including 365-day mortality, were similar between the low- and high-target groups.

In this study, higher CIs were observed in the high-target group up to 24 h after PAC insertion, after which CIs converged between the groups. These elevated CIs were achieved at the expense of higher noradrenaline dosages and VIS. The VIS were significantly higher at all time points in the high-target group, and converged at 48 h. This most likely reflects clinical management practices, as awakening attempts were typically initiated after 24 h, coinciding with reductions in sedation and overall haemodynamic stabilization. Pharmacological circulatory support was almost exclusively limited to noradrenaline, with only a small proportion of patients receiving additional inotropes.

Literature remains inconclusive regarding the effect of noradrenaline on cardiac output. A previous substudy of the BOX-trial reported increased CIs in the high-target group, primarily attributable to a greater venous return and higher heart rate, as stroke volume indices did not differ significantly between groups.^14,18^ Conversely, an RCT comparing noradrenaline and adrenaline performed in AMICS patients reported stable CIs during the first 12 h of noradrenaline administration,^19^ while an observational study in cardiac surgery patients observed that increasing noradrenaline dosages were associated with decreased cardiac output in the majority of patients.^20^ However, even though OHCA patients often present with lower cardiac output, resembling the haemodynamic profile of CS patients, cardiac output often recovers rapidly in survivors.^1^ An observational study in resuscitated patients demonstrated that cardiac output recovered within 24 h in most cases, with nearly all patients exhibiting normalization of cardiac output within 72 h, indicative of transient post-resuscitation myocardial dysfunction.^21^ In our cohort, CI recovered eventually in the high- as well as in the low-target group.

Interestingly, despite higher CIs, wedge pressures were found to be significantly higher in the high-target group. This may partly be explained by the higher administered fluids, as well as by the noradrenaline-induced vasoconstriction, which may cause increased venous return.^18^ Although SVR decreased in both groups over time, the high-target group consistently exhibited slightly higher values, reaching statistical significance after 24 h. Together, these factors could have raised filling pressures and have resulted in persistently higher PCWP values in the high-target group, thereby possibly placing additional stress on the post-arrest heart.

Importantly, lactate levels were similar between groups at all measured time points, and were cleared within 6 h in both groups, irrespective of differences in MAP and CI. Also, lactate levels were similar despite higher SvO_2_ in the high-target group at 6 and 12 h. This may indicate that reduced blood flow allowed for increased oxygen extraction by the tissues, thereby maintaining adequate oxygen delivery and resulting in similar lactate levels across groups.^22^

Similarly to the observations in the total BOX-population, mortality rates were similar between the low- and high-target groups. However, in this subpopulation of OHCA-AMICS patients, 365-day mortality rates were numerically lower in the low-target group, although this difference did not reach statistical significance (pLogrank = 0.12), potentially due to the small sample size and limited statistical power. Observed relative reductions in mortality and RRT in the low-target group (relative risk 0.78 [95% CI, 0.55–1.12]) are consistent with the power calculations for the NORshock trial, which tests two noradrenaline-mediated MAP targets (50–55 mmHg vs. >65 mmHg).^15^ It was designed to detect a 10% absolute (22% relative) reduction in the primary composite endpoint of all-cause mortality and need for RRT with 80% power. The primary endpoint rate was assumed to be 45.9% in the high MAP-target group.

The rationale for this potential benefit of a lower MAP-target in AMICS patients is based on the physiological effects of noradrenaline administration. Noradrenaline, while frequently administered to increase blood pressure and perfusion pressure, is also associated with potentially adverse (cardiac) effects. Its use is associated with elevated afterload, increased myocardial oxygen consumption, and a potential decrease of myocardial blood flow due to its vasoconstrictive effects on the coronary arteries.^5,7–9^ These effects might be particularly detrimental in patients who have recently experienced an AMI. Hypothetically, reducing noradrenaline dosages and thereby allowing for permissive hypotension, may improve clinical outcomes.

It was long thought that targeting lower MAPs in cardiac arrest patients was particularly hazardous due to the potential risk of compromised cerebral perfusion. The results of the BOX-trial contributed to recent updates of the European and American resuscitation guidelines, in which the MAP recommendations have been lowered to >60–65 and >65 mmHg, respectively.^23,24^ The present subanalysis extends these findings by suggesting that targeting lower MAPs is also safe in resuscitated AMICS patients, both in terms of mortality rates and a distribution of CPC and mRS scores that favoured the low-target group. These results suggest that lower MAP targets do not adversely affect neurological outcomes in this patient population. Importantly, while the current study indicates that lower MAP targets are safe, the NORshock trial is designed to target an even lower MAP target of 50–55 mmHg. Possibly, reducing noradrenaline further might yield additional better outcomes, even in this high-risk group of AMICS patients with cardiac arrest.

Our findings challenge the assumption that higher MAP and CI in OHCA-AMICS patients, achieved through increased noradrenaline administration, necessarily translate into improved tissue perfusion and outcomes. This underscores the importance of re-evaluating current noradrenaline-driven haemodynamic targets in this population.

## Limitations

This study has several limitations coherent with its descriptive design. First, given the neutral results of the BOX-trial, the numerically lower mortality in the low-target group in this subanalysis should be considered as hypothesis-generating. Second, due to the nature of the inclusion procedures of the BOX-trial, time points from OHCA to PCI at the catheterization laboratory, and subsequently to randomization in the ICU, vary between patients. As a result, the timing of haemodynamic and laboratory measurements ‘at admission’ differs among patients. Also, although this subanalysis benefited from the addition of laboratory values from electronic patient records to those collected at predefined time points, some of these could reflect laboratory values obtained by clinical indication. Third, it is important to note the potential for survival bias, as measurements were only available for patients who survived to the measuring point. Lastly, the current substudy is underpowered compared to the original sample size calculation to draw firm conclusions.

## Conclusion

In this substudy of the BOX-trial, higher MAPs achieved with noradrenaline resulted in higher CIs and elevated SvO_2_. However, lactate levels and clinical outcomes were comparable, while PCWP was higher, suggesting that higher noradrenaline-mediated MAP targets and CIs do not improve tissue perfusion or clinical outcomes and may place greater stress on the heart and lungs.

## Supplementary Material

zuag045_Supplementary_Data

## Data Availability

The data that support the findings of this study are available from the corresponding author upon reasonable request.
