# Supplementary material for: The impact of blood pressure targets on haemodynamics in patients with cardiogenic shock caused by infarction-related out-of-hospital cardiac arrest: a substudy of the BOX-trial
Source: Eur Heart J Acute Cardiovasc Care. 2026 May 11;15(8):584–93. doi: 10.1093/ehjacc/zuag045 (PMC13525482; doi:10.1093/ehjacc/zuag045)
Supplement: Supplementary file 1 [file zuag045_supplementary_data.docx]

Supplemental Table 1 – Primary endpoint and key in-and exclusion criteria of the BOX and NORshock trial

BOX-trial:

| Primary endpoint: a composite of death from any cause, or discharge from the hospital with a Cerebral Performance Category (CPC) of 3 or 4 within 90 days after randomization | |
| --- | --- |
| Inclusion criteria: | Exclusion criteria: |
| - Adult patients ≥18 years - OHCA of presumed cardiac cause - Sustained Return of Spontaneous Circulation >20 minutes - Unconsciousness (GCS <9) after sustained ROSC | - Unwitnessed asystole - Suspected acute intracranial bleeding or stroke - In-hospital cardiac arrest |

NORshock trial:

| Primary endpoint: composite of 30-day mortality and renal replacement therapy | |
| --- | --- |
| Inclusion criteria: | Exclusion criteria: |
| 1. Acute myocardial infarction; STEMI or NSTEMI 2. Early revascularization by PCI 3. Cardiogenic shock, characterized by: I. a. Systolic blood pressure (SBP) <90 mmHg for > 30 minutes, OR   b. Use of drugs to maintain SBP > 90 mmHg at randomization   II. Clinical signs of impaired organ perfusion with at least one of the following criteria:     a. Altered mental status     b. Cold, clammy skin and extremities     c. Oliguria with urine output < 30 mL / hour     d. Serum lactate > 2.0 mmol/L III. Clinical signs of pulmonary congestion | - Resuscitation > 30 minutes - Mechanical cause of cardiogenic shock (e.g. papillary muscle rupture, ventricular septal rupture) - Onset of shock > 12 hours - Imminent need for mechanical circulatory support (i.e. ECPR) - Women < 45 year |

*ECPR = Extracorporeal Cardiopulmonary Resuscitation; PCI = percutaneous coronary intervention; (N)STEMI = (non-) ST-elevation myocardial infarction*

Supplemental Table 2 – Baseline characteristics NORshock qualifying patients

| **Characteristic** | **Overall** | **NORshock eligible** | **Not eligible** | **p-value** |
| --- | --- | --- | --- | --- |
|  | **N = 789** | **N = 258** | **N = 531** |  |
| **Baseline characteristics** | | | | |
| Age, years, median (IQR) | 63 (13) | 63 (11) | 62 (14) | 0.44^1^ |
| Sex, male - n (%) | 637 (81%) | 217 (84%) | 420 (79%) | 0.094^3^ |
| Body mass index, n (%) |  |  |  | 0.085^4^ |
| Underweight, BMI < 18.5 | 14 (1.8%) | 1 (0.4%) | 13 (2.5%) |  |
| Normal Weight, BMI 18.5-24.9 | 274 (36%) | 99 (40%) | 175 (34%) |  |
| Overweight, BMI 25-29.9 | 295 (38%) | 94 (38%) | 201 (39%) |  |
| Obese, BMI ≥ 30 | 185 (24%) | 55 (22%) | 130 (25%) |  |
| **Comorbidities** | | | | |
| Heart Failure, no. (%) | 137 (17%) | 18 (7.0%) | 119 (22%) | <0.001^3^ |
| Diabetes, no. (%) | 110 (14%) | 35 (14%) | 75 (14%) | 0.83^3^ |
| Ischemic heart disease, no. (%) | 172 (22%) | 46 (18%) | 126 (24%) | 0.056^3^ |
| **OHCA characteristics** | | | | |
| Witnessed arrest, no. (%) | 672 (85%) | 218 (84%) | 454 (86%) | 0.67^3^ |
| Bystander cardiopulmonary resuscitation, no. (%) | 679 (88%) | 208 (82%) | 471 (90%) | 0.002^3^ |
| Time to ROSC, median (IQR) | 18 (12, 26) | 16 (11, 22) | 20 (12, 30) | <0.001^2^ |
| More than 30 minutes to ROSC, no. (%) | 121 (16%) | 0 (0%) | 121 (24%) | <0.001^3^ |
| **Admission characteristics** | | | | |
| LVEF at arrival, median (IQR) | 35 (25, 45) | 35 (25, 45) | 35 (20, 48) | 0.60^2^ |
| Lactate at arrival, mmol/L, median (IQR) | 5.0 (2.9, 7.9) | 4.4 (2.4, 7.6) | 5.3 (3.1, 8.2) | 0.007^2^ |
| Glucose at arrival, mmol/L, median (IQR) | 13.2 (10.3, 17.0) | 13.3 (10.5, 16.8) | 13.1 (10.1, 17.1) | 0.89^2^ |
| SCAI class at admission, n (%) |  |  |  | 0.12^3^ |
| SCAI class B/C | 249 (32%) | 82 (32%) | 167 (31%) |  |
| SCAI class D | 236 (30%) | 88 (34%) | 148 (28%) |  |
| SCAI class E | 304 (39%) | 88 (34%) | 216 (41%) |  |
| ST-segment elevation ECG, no. (%) | 350 (45%) | 182 (71%) | 168 (32%) | <0.001^3^ |
| Acute percutaneous intervention, no. (%) | 336 (47%) | 258 (100%) | 78 (17%) | <0.001^3^ |

*^1^* Welch two-sample t-test

*^2^* Wilcoxon rank sum test

*^3^* Pearson’s Chi-squared test

^4^ Fisher’s exact test

Supplemental Table 3 – Hemodynamic, tissue perfusion and metabolic measurements

|  | | **Time points (in hours) – 0 indication time of randomization** | | | | | | |
| --- | --- | --- | --- | --- | --- | --- | --- | --- |
| **Parameter** | **Intervention** | **At admission** | **0** | **6** | **12** | **24** | **36** | **48** |
| **Noradrenaline, µg/kg/min, (median with bootstrapped 95%CI)** | Low |  | 0.00 (0.00-0.00) | 0.09 (0.06-0.12) | 0.09 (0.07-0.13) | 0.12 (0.10-0.14) | 0.09 (0.07-0.14) | 0.09 (0.06-0.12) |
|  | High |  | 0.00 (0.00-0.00) | 0.17 (0.13-0.21) | 0.20 (0.17-0.24) | 0.24 (0.21-0.30) | 0.21 (0.17-0.26) | 0.17 (0.12-0.19) |
|  | P-value |  | 0.45 | < 0.001 | < 0.001 | < 0.001 | < 0.001 | < 0.01 |
| **Vasoactive inotropic score, (median with bootstrapped 95%CI)** | Low |  | 0.0 (0.0-0.0) | 6.0 (4.5-9.0) | 9.0 (7.3-13.0) | 10.7 (7.0-13.2) | 6.3 (4.0-10.3) | 5.8 (2.5-8.6) |
|  | High |  | 0.0 (0.0-0.0) | 17.1 (14.0-21.1) | 20.8 (17.0-24.8) | 22.9 (17.5-28.0) | 17.1 (10.0-24.0) | 8.1 (5.0-14.0) |
|  | P-value |  | 0.38 | < 0.001 | < 0.001 | < 0.001 | < 0.001 | 0.037 |
| **Mean arterial blood pressure, mm Hg** | Low | 77.4 (73.0-81.7) | 67.2 (63.2-71.2) | 64.9 (63.7-66.0) | 65.3 (64.2-66.5) | 66.8 (65.6-68.0) | 69.2 (67.5-70.8) | 75.3 (72.3-78.3) |
|  | High | 77.2 (72.5-81.9) | 78.2 (74.3-82.1) | 75.1 (73.9-76.2) | 76.0 (74.9-77.2) | 78.6 (77.3-79.8) | 78.6 (76.9-80.3) | 82.9 (79.7-86.1) |
|  | Difference | -0.2 (-6.6-6.2) | 11.0 (5.4-16.6) | 10.2 (8.5-11.8) | 10.7 (9.1-12.3) | 11.8 (10.1-13.5) | 9.4 (7.1-11.8) | 7.6 (3.2-12.0) |
|  | P-value | 0.95 | < 0.001 | < 0.001 | < 0.001 | < 0.001 | < 0.001 | < 0.001 |
| **Lactate, mmol per liter** | Low | 4.18 (3.70-4.73) | 2.88 (2.54-3.26) | 1.56 (1.40-1.73) | 1.48 (1.33-1.64) | 1.30 (1.19-1.42) | 1.34 (1.23-1.45) | 1.32 (1.22-1.43) |
|  | High | 4.28 (3.78-4.86) | 2.98 (2.62-3.38) | 1.69 (1.52-1.88) | 1.65 (1.48-1.84) | 1.39 (1.27-1.53) | 1.38 (1.27-1.51) | 1.42 (1.30-1.54) |
|  | Ratio | 1.02 (0.86-1.22) | 1.03 (0.87-1.24) | 1.08 (0.93-1.26) | 1.12 (0.96-1.30) | 1.07 (0.94-1.21) | 1.03 (0.92-1.17) | 1.07 (0.96-1.21) |
|  | P-value | 0.78 | 0.71 | 0.29 | 0.15 | 0.31 | 0.57 | 0.22 |
| **pH** | Low | 7.22 (7.21-7.24) | 7.27 (7.25-7.28) | 7.35 (7.34-7.36) | 7.36 (7.35-7.38) | 7.37 (7.37-7.38) | 7.38 (7.37-7.39) | 7.39 (7.38-7.40) |
|  | High | 7.22 (7.20-7.24) | 7.26 (7.25-7.28) | 7.33 (7.32-7.35) | 7.36 (7.35-7.37) | 7.37 (7.36-7.38) | 7.38 (7.37-7.39) | 7.39 (7.38-7.40) |
|  | Difference | 0.00 (-0.03-0.02) | -0.01 (-0.03-0.02) | -0.02 (-0.03-0.00) | -0.01 (-0.02-0.01) | -0.01 (-0.02-0.01) | 0.00 (-0.01-0.01) | 0.00 (-0.02-0.01) |
|  | P-value | 0.83 | 0.65 | 0.05 | 0.48 | 0.35 | 0.94 | 0.86 |
| **Base Excess** | Low | -7.15 (-8.09--6.20) | -5.40 (-6.31--4.49) | -4.16 (-4.83--3.48) | -3.07 (-3.71--2.43) | -2.27 (-2.89--1.66) | -1.68 (-2.37--1.00) | -0.58 (-1.28-0.12) |
|  | High | -7.96 (-8.92--7.01) | -6.09 (-7.00--5.17) | -4.28 (-4.95--3.62) | -3.33 (-3.97--2.69) | -2.65 (-3.27--2.04) | -1.48 (-2.17--0.80) | -0.62 (-1.32-0.09) |
|  | Difference | -0.82 (-2.16-0.53) | -0.68 (-1.97-0.61) | -0.13 (-1.08-0.82) | -0.26 (-1.17-0.64) | -0.38 (-1.24-0.48) | 0.20 (-0.77-1.17) | -0.03 (-1.03-0.97) |
|  | P-value | 0.23 | 0.30 | 0.79 | 0.57 | 0.39 | 0.68 | 0.9 |

|  | | **Time points (in hours) - 0 indicating time of PAC insertion** | | | | | | |
| --- | --- | --- | --- | --- | --- | --- | --- | --- |
| **Parameter** | **Intervention** | **At admission** | **0** | **6** | **12** | **24** | **36** | **48** |
| **Cardiac index, liters per minute per m2** | Low |  | 1.50 (1.41-1.59) | 1.66 (1.57-1.75) | 1.91 (1.81-2.02) | 2.19 (2.08-2.30) | 2.70 (2.54-2.86) | 2.88 (2.70-3.06) |
|  | High |  | 1.77 (1.67-1.89) | 1.95 (1.84-2.07) | 2.07 (1.96-2.19) | 2.42 (2.30-2.55) | 2.84 (2.67-3.02) | 2.92 (2.75-3.11) |
|  | Ratio |  | 1.19 (1.09-1.29) | 1.18 (1.09-1.28) | 1.08 (1.00-1.17) | 1.11 (1.03-1.19) | 1.05 (0.97-1.15) | 1.02 (0.93-1.11) |
|  | P-value |  | < 0.001 | < 0.001 | 0.046 | 0.007 | 0.23 | 0.70 |
|  | Low |  | 64.7 (63.0-66.5) | 64.8 (63.5-66.1) | 66.7 (65.4-68.0) | 69.2 (67.9-70.5) | 70.3 (68.8-71.7) | 69.6 (67.6-71.6) |
| **SvO_2_ (%)** | High |  | 66.0 (64.2-67.7) | 66.7 (65.4-68.1) | 68.8 (67.5-70.1) | 70.1 (68.8-71.4) | 69.8 (68.4-71.3) | 67.7 (65.6-69.8) |
|  | Ratio |  | 1.24 (-1.25-3.73) | 1.95 (0.03-3.87) | 2.13 (0.31-3.95) | 0.95 (-0.87-2.77) | -0.43 (-2.46-1.60) | -1.91 (-4.82-1.00) |
|  | P-value |  | 0.33 | 0.047 | 0.022 | 0.31 | 0.68 | 0.20 |
|  | Low |  | 15.7 (14.5-17.0) | 14.2 (13.3-15.2) | 14.0 (13.1-14.9) | 14.1 (13.1-15.1) | 13.6 (12.5-14.8) | 13.9 (12.5-15.3) |
| **PCWP** | High |  | 17.8 (16.5-19.0) | 16.5 (15.5-17.5) | 16.2 (15.3-17.2) | 15.7 (14.7-16.7) | 14.5 (13.3-15.6) | 14.4 (13.0-15.8) |
|  | Difference |  | 2.0 (0.2-3.8) | 2.3 (0.9-3.6) | 2.2 (0.9-3.5) | 1.6 (0.1-3.0) | 0.8 (-0.8-2.4) | 0.5 (-1.5-2.4) |
|  | P-value |  | 0.028 | < 0.001 | 0.001 | 0.032 | 0.30 | 0.62 |
|  | Low |  | 11.8 (10.5-13.1) | 10.5 (9.8-11.2) | 10.4 (9.0-11.7) | 10.2 (9.2-11.1) | 10.6 (9.4-11.8) | 10.9 (8.8-13.0) |
| **CVP** | High |  | 10.9 (9.6-12.2) | 11.1 (10.4-11.8) | 11.9 (10.5-13.3) | 11.9 (10.9-12.8) | 10.2 (9.0-11.5) | 11.4 (9.2-13.6) |
|  | Difference |  | -0.86 (-2.69-0.97) | 0.59 (-0.42-1.60) | 1.54 (-0.41-3.49) | 1.69 (0.32-3.06) | -0.38 (-2.14-1.38) | 0.51 (-2.51-3.54) |
|  | P-value |  | 0.35 | 0.25 | 0.12 | 0.016 | 0.67 | 0.74 |
|  | Low |  | 1503 (1406-1600) | 1372 (1290-1454) | 1195 (1112-1280) | 1051 (988-1115) | 907 (842-973) | 840 (757-925) |
| **SVR** | High |  | 1561 (1461-1661) | 1350 (1265-1435) | 1250 (1163-1336) | 1127 (1062-1192) | 1000 (932-1067) | 971 (887-1056) |
|  | Difference |  | 58 (-82-198) | -22 (-140-96) | 54 (-67-174) | 76 (-15-167) | 99 (-2-187) | 131 (11-250) |
|  | P-value |  | 0.41 | 0.72 | 0.38 | 0.10 | 0.05 | 0.032 |
